# Transsacrococcygeal approach to ganglion impar block for treatment of chronic coccygodynia after spinal arachnoid cyst removal: A case report

**DOI:** 10.1097/MD.0000000000005010

**Published:** 2016-09-30

**Authors:** Young Deog Cha, Chun Woo Yang, Jung Uk Han, Jang Ho Song, WonJu Na, Sora Oh, Byung-Gun Kim

**Affiliations:** Department of Anesthesiology and Pain Medicine, Inha University School of Medicine, Inha University Hospital, Incheon, South Korea.

**Keywords:** case report, coccygodynia, ganglion impar block, neurolysis, transsacrococcygeal junction

## Abstract

**Background:**

Coccygodynia is a pain in the region of the coccyx that radiates to the sacral, perineal area. The cause of the pain is often unknown. Coccygodynia is diagnosed through the patient's past history, a physical examination, and dynamic radiographic study, but the injection of local anesthetics or a diagnostic nerve blockade are needed to distinguish between somatic, neuropathic, and combined pain. Ganglion impar is a single retroperitoneal structure made of both paravertebral sympathetic ganglions. Although there are no standard guidelines for the treatment of coccygodynia, ganglion impar blockade is one of the effective options for treatment.

**Methods:**

Here, we report a 42-year-old female patient presenting with severe pain in the coccygeal area after spinal arachnoid cyst removal.

**Results:**

Treatment involved neurolysis with absolute alcohol on the ganglion impar through the transsacrococcygeal junction. Pain was relieved without any complications.

**Conclusion:**

Our case report offers the ganglion impar blockade using the transsacrococcygeal approach with absolute alcohol can improve intractable coccydynia.

## 1 Introduction

Coccygodynia is a condition characterized by pain and tenderness in the tailbone or perianal area. It occurs when the pain associated with a fracture of the tailbone varies from a dull to a severe sharp pain. Patients with coccygodynia typically complain of pain when sitting on hard chair and during defecation and finger insertion in the rectum. The force applied to the coccyx seriously affects their daily life.^[1,2]^ Coccygodynia has many causes. It may be posttraumatic, beginning after a fracture or contusion or after difficult vaginal delivery. Chronic microdamage to the coccyx from an incorrect posture or bursitis on the coccygeal periosteum is also part of the pathogenesis. Moreover, coccygodynia is related to the body mass index (BMI), and the cause is often unknown.^[2,3]^ There are no standard treatment guidelines despite the existence of many modalities, including physical therapy, local infiltration of local anesthetics and steroids, neurolysis of the sacral nerve root, and caudal epidural block. Furthermore, coccygectomy is not recommended due to problems, such as high rate of infection.^[4]^ In the present case, a favorable result was obtained by using alcohol for sympatholysis of the ganglion impar in a patient who had been suffering from chronic coccygodynia for 6 years.

## 2 Case report

**Figure 1:**
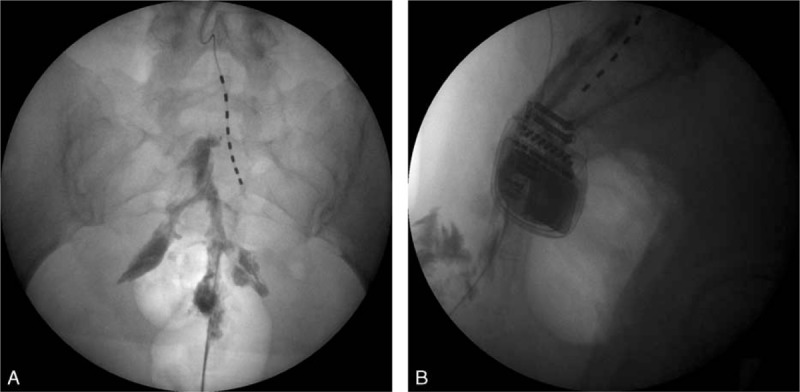
The A-P (A), and lateral view (B) after contrast media injection during caudal epidural block.

**Figure 2:**
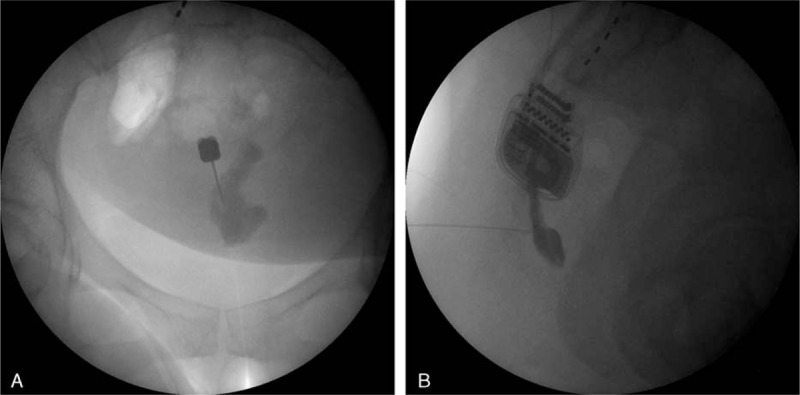
The A-P (A), and lateral view (B) after contrast media injection during ganglion impar block.

A 42-year-old female visited our pain clinic because of severe pain in the coccygeal area. Her weight was 132 lb (60 kg) and height 62.25 inch (158 cm), with a calculated body mass index (BMI; kg/m^2^) of 24. The patient had undergone spinal arachnoid cyst removal 6 years earlier, and the coccygeal pain had been severe since then. The patient was taking oral opioid medication (oxycodone hydrochloride, 20 mg/day). As several caudal epidural blocks and L4–5 epidural blocks had failed to control the pain, the patient had received a spinal cord stimulator implant 3 years earlier. The patient described her coccygeal pain as burning, stabbing, heavy, and pressing with a visual analog score (VAS) of 9/10. There was pain from palpation, and the patient could not assume a sitting position for >5 minutes. The physical examination revealed no sensory deterioration. Lab tests were normal and there were no fractures or dislocations on the sacrococcygeal plain radiography. The curve, length, and shape of the coccyx were normal. There were no abnormal findings on computed tomography and magnetic resonance imaging scans, which included the abdomen and pelvis. At the time of her initial outpatient visit, a diagnostic caudal epidural block was performed (Fig. 1A and B). Twenty milliliters of 1% lidocaine was injected into the distal caudal epidural space through the sacral hiatus. The patient was monitored clinically. For 2 days after the block, the VAS decreased from 9/10 to 5/10. We performed a second caudal epidural block 4 days after her first visit. The result was the same. Therefore, we considered the pain to be sympathetically maintained, and the patient was advised to undergo a C-shaped image intensifier guided blockage of the ganglion impar with alcohol. Three days later, a test blockage was performed using 6 mL of 1% lidocaine, and the pain temporarily decreased. After informed consent, we performed a blockage of the ganglion impar with 99.9% alcohol 7 days later. For the blockage of the ganglion impar, the patient was placed in the left decubitus position on a radioparent table. Blood pressure, heart rate, and pulse oximetry were monitored throughout the procedure. The blockage of the ganglion impar was not performed with the original technique described by Plancarte et al, but with a transsacrococcygeal approach technique^[5]^. The site of the needle insertion was located by palpating the sacral cornu and using a C-shaped image intensifier following chlorohexidine aseptic preparation. Under guidance from the C-shaped image intensifier in the AP position, a 22-gauge, 5-cm block needle was inserted through the skin by piercing the dorsal sacrococcygeal ligament at the midline. Using the loss of resistance method, the needle was advanced through the vertebral disc until the needle tip was placed anteriorly to the ventral sacrococcygeal ligament. The position of the needle tip was confirmed by injecting 3 mL of contrast medium into the retroperitoneal space. On AP and lateral images, the spread of contrast medium gave an “apostrophe” appearance (Fig. 2A and B). A therapeutic neurolytic block was then performed with 4 mL of 99.9% alcohol. No adverse events including hypotension, bleeding, drug allergy, or seizure occurred during the procedure. After the blockade, the patient was monitored in the same position for 2 hours. The patient perceived the VAS to have decreased from 9/10 to 3/10, and she could sit at ease. The patient was discharged from the clinic on the day and the pain reduction was maintained for 2 weeks. At present, 3 months after the treatment, the patient has maintained a VAS of 2/10 and has experienced a satisfactory outcome in her daily life and work, including being able to sit for >1 hour as compared to <5 minutes before the treatment. The patient has not taken any analgesics or opioids.

## 3 Discussion

Coccygodynia is a pain in the region of the coccyx that radiates to the sacral, perineal area. The cause of the pain is often unknown. Coccygodynia is 5 times more prevalent in women than in men. Women are more exposed than men as their sacrum and coccyx are located more posterior.^[1,2]^ A fall onto the buttocks may cause contusion, fracture, or dislocation of the tailbone. The incidence of a sacrococcygeal ligament injury during vaginal delivery can also cause pain. As the coccyx is mobile and supported by the sacrococcygeal ligament, sprains are more likely to occur than fractures. Microtraumas from inadequate body positioning while seated can also cause chronic sprain of the coccyx.^[3]^ Coccygodynia is closely related to the body mass index.^[4]^ Symptoms include the development of a musculoskeletal disease and referred pain from lumbar pathologies. However, careful differential diagnosis is needed as the cause of the pain can often be idiopathic.^[6,7]^ The third, fourth, and fifth sacral nerves are related to coccygodynia, and the sympathetic nervous system (ganglion impar) can cause hyperalgesia and allodynia.^[7]^ The pain may be caused by a musculo-neuro-fascial disruption of the joint (somatic pain or neuropathic pain) and by the stimulation of distant organs (referred pain).^[4]^ Coccygodynia is diagnosed through the patient's past history, a physical examination, and dynamic radiographic study, but the injection of local anesthetics or a diagnostic nerve blockade are needed to distinguish between somatic, neuropathic, and combined pain.^[6,7]^

Hyperalgesia of sympathetically maintained pain is believed to be caused by the sensitization of the central pain-signaling neurons to the mechanoreceptor input. Sympathetic signs, such as a decrease in skin temperature, hypohydrosis, and an increase in muscle tone, do not assist in the diagnosis as they may or may not appear. The most definitive method for the diagnosis of sympathetically maintained pain is a sympathetic block.^[8]^ Epidural blocks with 1% lidocaine are used for the differential diagnosis of both the systemic and sympathetic nerves. If the pain relief subsides within 2 hours, the pain is somatosensory. If it lasts longer, the pain is sympathetic.^[8]^ Caudal epidural blocks are preferred for the differential diagnosis of coccygodynia as they can be performed in outpatient clinics. Only local anesthetics are required, so that the method presents fewer complications while allowing us to differentiate between somatosensory and sympathetic pain perfectly.^[8]^ In this present case, the pain was reduced with 2 caudal epidural blocks using only local anesthetics. Therefore, we considered the pain to be sympathetically maintained and planned to perform a C-shaped image intensifier guided blockage of the ganglion impar.

The sympathetic trunk is composed of 21 or 22 sympathetic ganglions. The ganglion impar (or ganglion of Walther) is the midline caudal termination of the paravertebral sympathetic chains.^[9]^ The ganglion impar is a single retroperitoneal structure made of both sympathetic ganglions joined at the sacrococcygeal joint. It is found in a variable position in the precoccygeal space and may lie up to 1.9 cm from its classically described position as anterior to the sacrococcygeal joint. Its exact position on the coccyx is diverse, with variations observed from the level of the sacrococcygeal junction to below the midpoint of the coccyx. The ganglion supplies nociceptive and sympathetic fibers to the pelvic viscera. It is located mainly at the midline of the precoccygeal space, but can also sometimes be lateral to the midline on the left or right side.^[5]^

Plancarte started using ganglion impar blocks to treat perineal pain in 1990. It is an effective pain relief modality for perineal cancer pain, such as rectal cancer and cervical cancer.^[10,11]^ Perineal cancer pain, perianal hyperhidrosis, perianal pain, and coccygodynia are the main indications for ganglion impar blocks, and satisfactory results have been reported. The original technique described by Plancarte et al^[5]^ used a bent 22-gauge spinal needle introduced through the anococcygeal ligament and directed under fluoroscopic guidance to lie with its tip retroperitoneally at the sacrococcygeal junction. After confirmation of an “apostrophe” contrast medium pattern, local anesthetics or neurolytic agents are injected.^[10]^ However, this method presents a risk of inaccurate needle location and patients complain of pain during the procedure.^[10]^ A modified approach uses a needle entry point below the transverse process of the coccyx, but there is a potential risk of chemical neurolysis including motor, sexual, and bowel or bladder dysfunction as a result of sacral and coccygeal plexus damage due to the inadvertent spread of the neurolytic agent.^[12]^ In 1995, Wemm and Saberski^[13]^ described a “transsacrococcygeal ligament” approach. The latter is believed to cause less tissue damage and to be less painful, and it appears to be simpler to perform. For this reason, we performed a ganglion impar blockade using a safe transsacrococcygeal approach and the patient felt less pain and was more comfortable. Either the prone or lateral decubitus position can be chosen for the transsacrococcygeal approach. However, if the patient can assume a comfortable position and the operator is right handed, the lateral decubitus position is believed to make the procedure easier. Although we used the transsacrococcygeal approach, neurolytics were injected afterward to confirm that the spread of the contrast media had an “apostrophe” appearance, as in the original technique described by Plancarte et al.^[5]^

Repeated blockades of the ganglion impar with local anesthetics block the vicious circle of pain and can provide prolonged relief of coccygodynia.^[10]^ In cases of cancer and intractable pain, absolute alcohol^[14]^ or phenol^[15]^ can be injected for neurolysis. The neurolytics must be injected carefully after confirmation of the contrast media spread to the retroperitoneal space, as they do not always spread to the intended site. As the heavy use of neurolytics to improve the success rate of neurolysis can cause dysuria or dyschezia, much care is needed.^[5]^ Complications include infections, bleeding, and sacrococcygeal disc damage. However, there were no complications in this case.

## 4 Conclusion

This case report suggests that ganglion impar blockade using the transsacrococcygeal approach with absolute alcohol can improve intractable coccydynia. We performed this block using a 22-gauge, 5-cm block needle via the trans-sacrococcygeal approach where possible under fluoroscopic guidance. We have found this to be a simple and efficient approach to ganglion impar.
